# Rural-Urban Differences in Household Treatment-Seeking Behaviour for Suspected Malaria in Children at Bata District, Equatorial Guinea

**DOI:** 10.1371/journal.pone.0135887

**Published:** 2015-08-18

**Authors:** Maria Romay-Barja, Inma Jarrin, Policarpo Ncogo, Gloria Nseng, Maria Jose Sagrado, Maria A. Santana-Morales, Pilar Aparcio, Basilio Valladares, Matilde Riloha, Agustin Benito

**Affiliations:** 1 Centro Nacional de Medicina Tropical, Instituto de Salud Carlos III, Madrid, Spain; 2 Red de Investigación Colaborativa en Enfermedades Tropicales, RICET, Madrid, Spain; 3 Centro Nacional de Epidemiología, Instituto de Salud Carlos III, Madrid, Spain; 4 Centro de Referencia de Control de Endemias, Malabo, Equatorial Guinea; 5 Ministerio de Salud y Bienestar Social, Malabo, Equatorial Guinea; 6 Instituto Universitario de Enfermedades Tropicales y Salud Pública de Canarias, Tenerife, Spain; Centro de Pesquisa Rene Rachou/Fundação Oswaldo Cruz (Fiocruz-Minas), BRAZIL

## Abstract

**Background:**

Malaria remains a major cause of morbidity and mortality among children under five years old in Equatorial Guinea. However, little is known about the community management of malaria and treatment-seeking patterns. We aimed to assess symptoms of children with reported malaria and treatment-seeking behaviour of their caretakers in rural and urban areas in the Bata District.

**Methodology:**

A cross-sectional study was conducted in the district of Bata and 440 houses were selected from 18 rural villages and 26 urban neighbourhoods. Differences between rural and urban caregivers and children with reported malaria were assessed through the chi-squared test for independence of categorical variables and the t-Student or the non-parametric Mann-Whitney test for normally or not-normally distributed continuous variables, respectively.

**Results:**

Differences between rural and urban households were observed in caregiver treatment-seeking patterns. Fever was the main symptom associated with malaria in both areas. Malaria was treated first at home, particularly in rural areas. The second step was to seek treatment outside the home, mainly at hospital and Health Centre for rural households and at hospital and private clinic for urban ones. Artemether monotherapy was the antimalarial treatment prescribed most often. Households waited for more than 24 hours before seeking treatment outside and delays were longest in rural areas. The total cost of treatment was higher in urban than in rural areas in Bata.

**Conclusions:**

The delays in seeking treatment, the type of malaria therapy received and the cost of treatment are the principal problems found in Bata District. Important steps for reducing malaria morbidity and mortality in this area are to provide sufficient supplies of effective antimalarial drugs and to improve malaria treatment skills in households and in both public and private sectors.

## Introduction

Malaria is endemic in Equatorial Guinea with stable transmission [1] and remains the major cause of morbidity and mortality among children under five years of age [2].

An intensive malaria control programme was introduced on the island of Bioko in 2004 and it achieved large reductions in infection, anaemia and child mortality [3]. Since 2007, similar interventions have been implemented in the four mainland provinces, under the Equatorial Guinea Malaria Control Initiative (EGMCI). The strategy consisted mainly in indoor residual spraying in Litoral and Kie-Ntem provinces, and mass distribution of long-lasting insecticide treated nets in Centro Sur and Wele-Nzas provinces. Case management was improved in public services in all four provinces, through the distribution of free artemisinin-based combination therapy and other measures [4]. The initiative was largely funded by The Global Fund to Fight AIDS, Tuberculosis, and Malaria (GFATM), and it was implemented by the government of Equatorial Guinea, in collaboration with several international organizations. Despite the malaria control programme implemented in the mainland region of Equatorial Guinea, prevalence has remained extremely high in children under five years old (52.2%) [4].

It is well recognized that accessibility to anti-malaria interventions alone will not bring about the desired change. Several studies have demonstrated that compliance with anti-malaria interventions depends substantially on social, behavioural, and cultural factors [5,6]. In addition, factors such as vulnerability, economic constraints, and the inadequacy or unavailability of appropriate health services play an important role in explaining the health seeking behaviour of the population [7].

Early diagnosis and prompt treatment has been the cornerstone of malaria control. However, effective case management partly depends on early recognition of the disease and subsequent treatment-seeking behaviour by care providers [8]. Although there is evidence of treatment-seeking behaviour in highly endemic areas in sub-Saharan Africa, there is a large range of variation in treatment-seeking patterns [9]. Therefore, studies are needed at the local level to understand the dynamics of this disease in a specific context.

Little is known about the community management of malaria and treatment-seeking patterns in Equatorial Guinea. Previous behavioural studies have been mainly conducted in Bioko Island and focused on prevention activities like bed nets usage [3, 4]. In relation with household treatment-seeking behaviour, one study reported that 45.3% of the children did not seek treatment outside home and 22.8% looked for treatment the same day of the fever onset [10]. There is a real need to understand community perceptions and practices about malaria in Equatorial Guinea and especially in the mainland region, where most of the country population lives.

Several features distinguish urban from rural malaria [11]. Differences in malaria transmission rates and different risk factors may lead to different disease burdens [12]. Also, rural and urban populations differ in their cultural practices, socioeconomic and demographic characteristics, availability and accessibility to treatment sources, provision of basic infrastructure, and childhood nutritional status [13]. Differences between rural and urban populations should be taken into account to improve the efficacy and efficiency of control interventions.

In this context, the present study aimed to assess symptoms, treatment-seeking behaviour, treatment administered, timeliness and costs related to the last reported malaria episode in children up to 15 years old, in rural and urban areas in the Bata District.

## Materials and Methods

### Study area

This cross-sectional study was carried out in June–August 2013, in the Bata District, mainland region of Equatorial Guinea. It was part of a project aimed to provide baseline data on the prevalence of malaria, the molecular characteristics of *Plasmodium*, and the vectors for malaria transmission in the area. The project also aimed to provide information about the knowledge, practices, and attitudes of the targeted population.

The District of Bata, with a population of 244,264 inhabitants, is the largest district in the country, according to the latest national census (DHS, 2011)[14]. The proportion of the population living in urban areas increased from 27.1% in 1975 to 48.3% in 2003 (UNDP, 2006)[10]. The District´s public health facilities comprise a network of seven health centres, two rural and five urban, and one regional hospital located in the city of Bata. There are also private health facilities in the Bata District, including three health centres, two hospitals, and about 20 clinics, all in the urban area of Bata city. Drug stores and pharmacies are distributed throughout the rural and urban areas of the District.

The first line treatment for uncomplicated malaria in Equatorial Guinea is artesunate-amodiaquine (AS+AQ); the second line treatment is artemether/lumefantrine (AL), and for severe malaria, quinine is recommended. However, with the withdrawal of GFTAM funding in 2011, the EGMCI stopped its main activities and there were stock shortages of first line treatment since 2012 (MoH unpublished report).

### Study population, sampling, and data collection

A descriptive cross-sectional study was designed to determine whether caretakers were able to recognize malaria symptoms and to compare the knowledge, attitudes and treatment practices of rural and urban households related to the last reported malaria episode of children up to 15 years old in the District of Bata. Sampling was carried out with a multistage stratified cluster strategy. First, 18 rural villages, out of 70, and 26 urban neighbourhoods, out of 111, were randomly selected with probability proportional to size to better assure representativeness in the sample design. Adequate sample size was computed to be able to detect statistical significant differences between urban and rural respondents and also to improve precision using a power of 80%, 95% confidence level and assuming a knowledge about malaria symptoms of 50%. The EPIDAT software version 3.1 was used to calculate sample size. The allocation of this sample size into those for urban and rural areas was done using proportionate allocation method considering their population resulting in a sampling size of 440 households. Second, households were randomly selected from an updated census from each cluster provided by the head of the village or neighbourhood.

Household caregivers were identified in each house and asked about their treatment-seeking behaviour relating to the most recent malaria episode in a child under their care. Specifically, we obtained information on household social characteristics and the symptoms, treatment and treatment timelines, sources and costs related to the last reported malaria episode in a child up to 15 years old. Eight nursing students, in their final year of training, were recruited and trained for 5 days to administer the questionnaire. The questionnaire was previously tested and translated into the main local language, Fang. All care providers were given the option to be interviewed in Fang or Spanish, the two official languages of the country.

### Data analysis

Houses were classified by their structures as follows: low: all houses with soil floors; middle: houses with cement or wood floors, plank wood walls, and iron sheet roofs; and high: cement or tile floors, cement, plank wood or brick walls, and iron sheet or cement roofs. Drinking water was considered clean when it came from a well, from a communal source, or bottled water. Water from the river was considered dirty or unprotected.

A descriptive analysis of participants’ characteristics was carried out using frequency tables for categorical variables and mean and standard deviation or median and interquartile range for normally or not-normally distributed continuous variables, respectively. Differences in socio-demographic characteristics and treatment-seeking behaviours between rural and urban caregivers of children with reported malaria episodes were assessed through the chi-squared test for independence for categorical variables and the t-Student or the non-parametric Mann-Whitney test for normally or not-normally distributed continuous variables, respectively. P-values <0.05 were considered to be statistically significant. Data was doubly entered with EpiData software v.3.1, and analysed with IBM SPSS Statistics 22.Ink.

### Ethics statement

This study was approved by the Ministry of Health and Social Welfare of Equatorial Guinea and the Ethics Committee of the Spanish National Health Institute, Carlos III (CEI PI 22_2013-v3). Written informed consent for participation in the study was obtained from the caregivers interviewed and from the heads of the households.

## Results

### Characteristics of caregivers

A total of 440 caregivers were interviewed but 12 were withdrawn from the analysis as their questionnaires were incomplete. Of 428 caregivers were interviewed about the last malaria episode in a child under their care, 173 (40.4%) lived in rural area and 255 (59.6%) in urban Bata. Table 1 summarizes the socio-economic and demographic characteristics of caregivers and their households. Differences between rural and urban households are clear. Caregivers were older in rural than in urban areas, with a median age of 40 (IQR: 30–50; minimum:15; maximum:70) and 30 (IQR: 25–40; minimum:15; maximum: 66) years, respectively. In rural areas, 21.0% of caregivers were illiterate but only 11.4% in urban areas (p = 0.008), where they also completed a higher educational level. Males were the heads of households in both rural (75.1%) and urban areas (58.8%), (p = <0.001). Employment was the main source of income in both areas, but subsistence farming was the main source of income in 24.9% of the rural families and 2.0% of the urban ones. Most houses were of the medium structure, but more households in rural areas than in urban areas had soil floors (27.2% vs. 8.6%), no access to electricity (47.4% vs. 90%), or no access to clean water (29.5% vs. 1.2%). A total of 2,242 children were living in the 428 houses. In both areas, there was a median of 1 child under one year old per house (p = 0.401). The median number of children aged 1 to 15 years per house was 4 in rural and 3 in urban areas (p = 0.009).

**Table 1:** Socio-economic and demographic characteristics of caretakers and their households in Bata District.

	Rural		Urban		
	n = 173	%	n = 255	%	*P-Value*
**Sex**					
Male	3	1.7	4	1.6	
Female	170	98.3	251	98.4	0.895
**Age**					
15–24	23	13.3	63	24.7	
25–34	46	26.6	108	42.4	
35–44	34	19.7	47	18.4	
45–54	47	27.2	24	9.4	
55–64	19	11.0	11	4.3	
65+	4	2.3	2	0.8	<0.001
**Marital status**					
Widow	8	4.6	12	4.7	
Currently married	113	65.3	145	56.9	
Single	50	28.9	96	37.6	
Divorced	2	1.2	2	0.8	0.297
**Educational level**					
No formal education	20	11.5	16	6.3	
Primary School	88	50.9	76	29.8	
Secondary School	65	37.5	158	61.9	
University	0	0.0	5	2.0	<0.001
**Ethnicity**					
Fang	149	86.1	217	85.1	
Combe	11	6.4	20	7.8	
Bisio	5	2.9	2	0.8	
Ndowe	8	4.6	16	6.3	0.305
**Main source of money**					
Agriculture	43	24.9	5	2.0	
Fishing	2	1.2	2	0.8	
Hunting	3	1.7	0	0.0	
Employee	60	34.7	146	57.3	
Business	58	33.5	88	34.5	
Other	7	4.0	14	5.5	<0.001
**House structure**					
Low	47	27.2	22	8.6	
Medium	107	61.8	159	62.4	
High	19	11.0	74	29.0	<0.001
**Acces to light**					
Yes	91	52.6	232	91.0	
No	82	47.4	23	9.0	<0.001
**Acces to clean water**					
Yes	122	70.5	252	98.9	
No	51	29.5	3	1.2	<0.001
**Latrine**					
Yes	149	86.1	243	95.3	
No	24	13.9	12	4.7	0.001
**Radio**					
Yes	139	80.3	185	72.5	
No	34	19.7	70	27.5	0.065
**TV**					
Yes	140	80.9	241	94.5	
No	33	19.1	14	5.5	<0.001

### Characteristics of surveyed children and symptoms

The characteristics of the children with reported malaria and the features of their last malaria episode are shown in Table 2. The percentage of males was slightly higher in rural than in urban areas (56.6% vs. 48.6%) and most of children were between 1–5 years old in both areas (61.3% vs. 56.9%). Malaria episodes had occurred more recently among rural children: 52.0% of rural children had a malaria episode during the prior two weeks in compared to 35.3% of urban children (p = 0.001).

**Table 2:** Characteristics of the children with reported malaria and the features of their last malaria episode in Bata District.

	Area				
	Rural		Urban		*P-Value*
	n	%	n	%	
**Time since last malaria episode**					
Now	13	7.5	5	2.0	
Last week	11	6.4	11	4.3	
Between one and two weeks	66	38.2	74	29.0	
Two weeks or +	83	48.0	165	64.7	0.001
**Sex of child**					
Male	98	56.6	124	48.6	
Female	75	43.4	131	51.4	0.103
**Relationship with household head**					
Son/Daugther	63	36.4	134	52.5	
Grandchild	77	44.5	81	31.8	
Nephew	15	8.7	16	6.3	
Great grandchild	4	2.3	4	1.6	
Brother/Sister	1	0.6	1	0.4	
Missing	13	7.5	19	7.5	0.041
**Age group (years)**					
< 1	20	11.6	41	16.1	
1–5	106	61.3	145	56.9	
> 5	47	27.2	69	27.1	0.403
**Who took the child outside for treatment?** *					
Father	6	4.4	10	4.4	
Mother	102	74.5	179	78.9	
Grandmother	17	12.4	25	11.0	
Father and mother	2	1.5	6	2.6	
Other	8	5.8	5	2.2	
Don´t know	2	1.5	2	0.9	0.503
**Treatment timelines** *					
Less than 24 hours	19	13.9	50	22.0	
1 day	30	21.9	80	35.2	
2 days	30	21.9	54	23.8	
3 days	16	11.7	23	10.1	
4 days	9	6.6	3	1.3	
5 days or more	13	9.5	9	4.0	
Don´t know	20	14.6	8	3.5	<0.001

*Information available for children who received treatment outside

**Fig 1:**
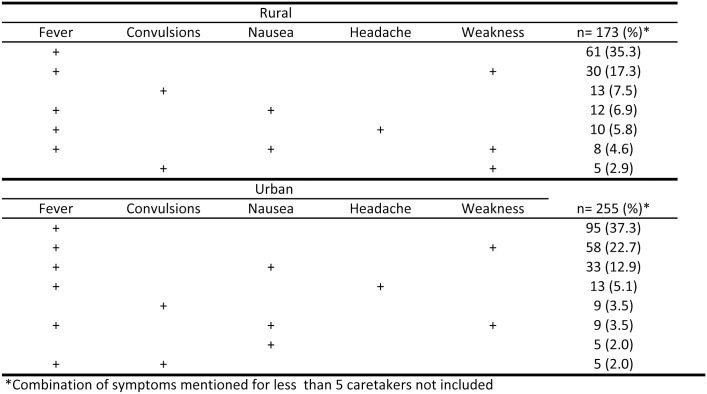
Multiple signs and symptoms mentioned by caretakers of children with reported malaria in Bata District.

The two malaria symptoms most often identified in children by rural and urban caretakers were fever (identified by 81.5% of rural and 90.2% of urban caretakers) and weakness (identified by 32.9% of rural and 31.8% of urban caretakers). The third most common symptom identified was nausea (21.6%) by urban caretakers and either convulsions (15.0%) or nausea (15.0%) by rural caretakers. When looking at symptoms according to age of children, a similar pattern to that observed overall was found. Most symptoms were not reported alone, Fig 1 shows the multiple signs and symptoms mentioned by caretakers. The two symptoms most mentioned in both areas were fever alone and fever and weakness together. The second symptom most mentioned alone in both areas was convulsions; this symptom was more frequently reported in rural areas (7.5%) than in urban areas (3.5%).

### Treatment-seeking behaviour

**Fig 2:**
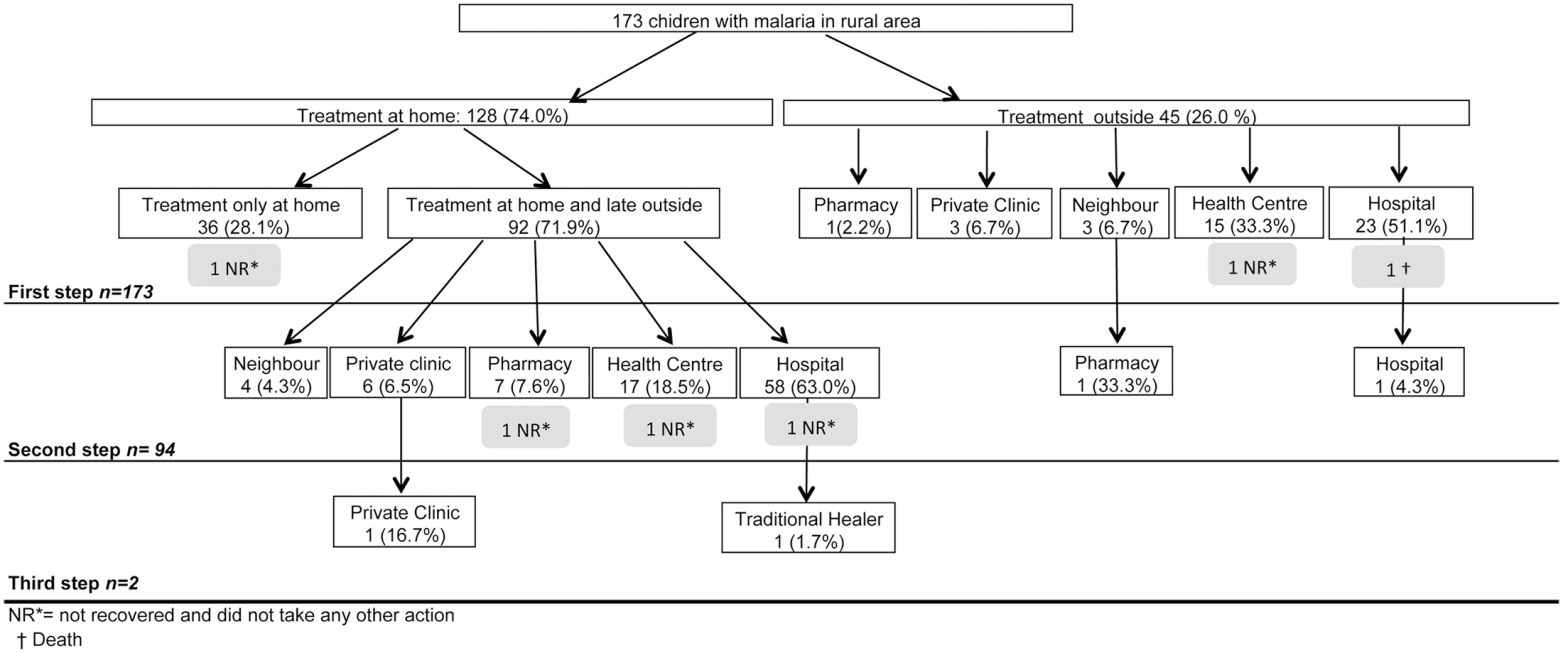
Treatment-seeking behaviours of caretakers for children with reported malaria in rural area of Bata District.

**Fig 3:**
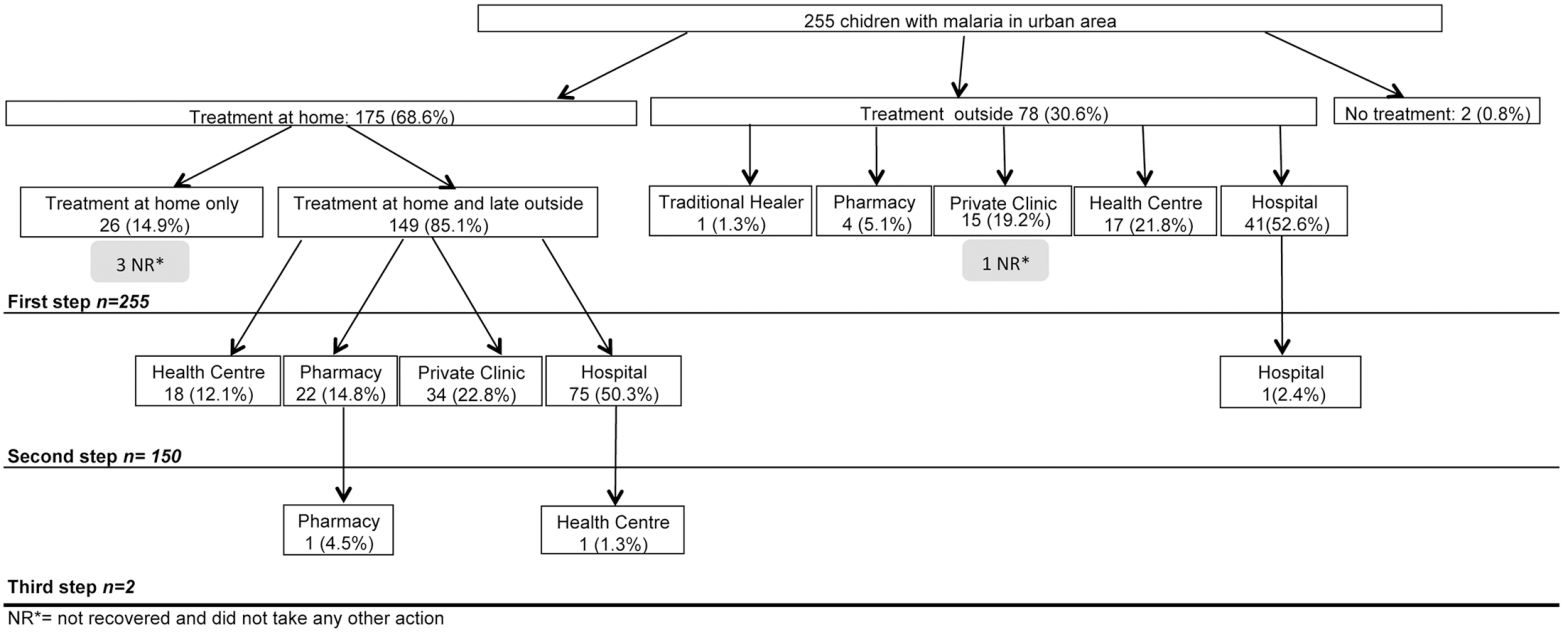
Treatment-seeking behaviours of caretakers for children with reported malaria in urban area of Bata District.

Figs 2 and 3 show treatment-seeking behaviours of caretakers for children with reported malaria in rural and urban areas, respectively. Overall, the three steps taken were significantly different between urban and rural respondents (all p<0.05).

Treatment at home was the first option most mentioned for both urban and rural caretakers of a child with reported malaria. The rural caretakers were more likely than urban caretakers to opt for home treatment (rural 74.0% vs. urban 68.6%). Also, rural children received treatment only at home (28.1%) more frequently than urban children (14.9%). Caretakers reported that, among the children treated only at home, 97.2% and 88.5% recovered in rural and urban areas, respectively.

**Fig 4:**
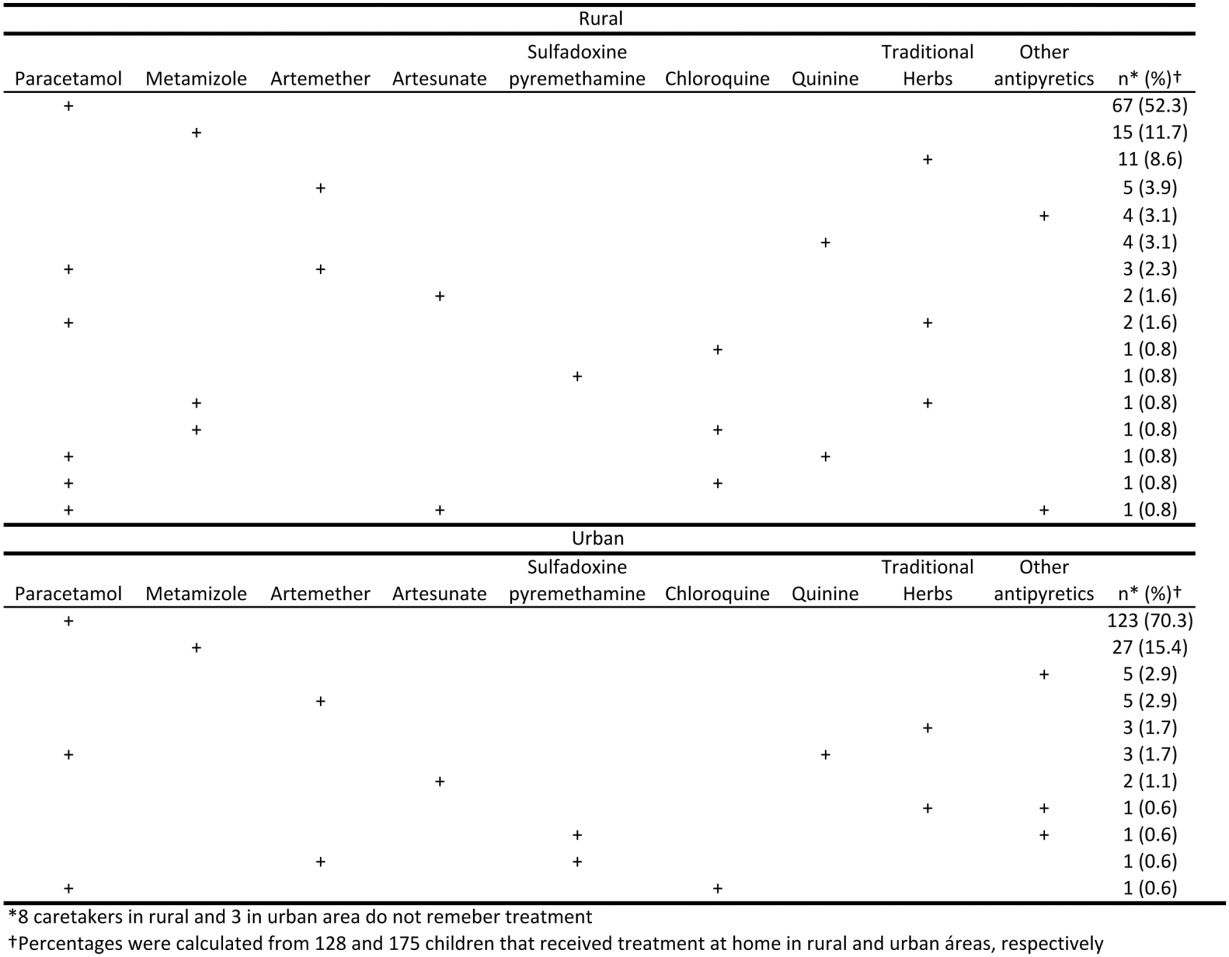
Drugs administered for children that received treatment at home in Bata District.

Treatment more frequently received at home was paracetamol (58.6% rural vs. 72.6% urban) followed by metamizol (13.3% vs. 15.4%). A traditional herbal treatment, *Nfoo* and *Eku*, was the third option for rural caretakers (10.9%) and the fifth option for urban caretakers (2.3%). Antimalarial drugs were given to 26.6% of rural children treated at home, but only 10.3% of urban children. The conventional antimalarial administered most often at home in both areas was artemether (6.3% rural vs. 3.4% urban). When looking at drugs administered by age of children, a similar pattern to that observed overall was found. Most treatments were not administered alone, Fig 4 shows combinations of treatments administered to children treated at home.

When the children were taken to a health service as a first step in seeking treatment, the most common first options were a hospital (51.1% rural vs. 52.6% urban) or a Health Centre (HC) (33.3% rural vs. 21.8% urban). The private clinic was also an important first option for urban caretakers (19.2%), but not for rural caretakers (6.7%). When the first step was treatment outside the home, caretakers reported that 91.1% of rural children and 97.4% of urban recovered.

Among the children that received treatment at home at first step, 71.9% of rural and 85.1% of urban children received a second step of treatment outside the home. In both areas hospital was the first option, particularly in rural areas (63.0%). The second option in rural areas was the HC (18.5%), and the second option in urban Bata was a private clinic (22.8%).

The treatments children received outside the home were most often artemether monotherapy (29.9% rural vs 33.5% urban) and paracetamol (26.3% rural vs 28.6% urban), regardless of where they went to seek a cure. The third most common treatment was sulfadoxine/pyrimethamine (SP) for 17.6% of urban and 13.9% of rural children. This treatment was administered most often in urban HCs and hospitals. Table 3 shows the drugs administered and the treatment places for children that received treatments outside the home in the Bata District. AS+AQ was the third treatment most administrated in pharmacies but was almost non-existent among private clinics and hospitals.

**Table 3:** Drugs administered and the treatment places for children that received treatment outside the home in the Bata.

	Pharmacy		Private Clinic		Health Centre		Hospital	
	Rural n = 8 (%)	Urban n = 26 (%)	Rural n = 9 (%)	Urban n = 49 (%)	Rural n = 32 (%)	Urban n = 35 (%)	Rural n = 81 (%)	Urban n = 116 (%)
Paracetamol	5 (62.5)	9 (34.6)	2 (22.2)	10 (20.4)	5 (15.6)	12 (34.3)	20 (24.7)	32 (27.6)
Artemether	2 (25.0)	9 (34.6)	2 (22.2)	12 (24.5)	4 (12.5)	11 (31.4)	31 (38.3)	43 (37.1)
Sulfadoxine/pyremethamine	1 (12.5)	2 (7.7)	1 (11.1)	4 (8.2)	2 (6.3)	6 (17.1)	15 (18.5)	27 (23.3)
AS+AQ	2 (25.0)	4 (15.4)	-	2 (4.1)	2 (6.3)	6 (17.1)	4 (4.9)	8 (6.9)
Chloroquine	-	-	1 (11.1)	1 (2.0)	1 (3.1)	1 (2.9)	-	3 (2.6)
Quinine	-	1 (3.8)	-	6 (12.2)	2 (6.3)	-	6 (7.4)	13 (11.2)
Artesunate (AS)	1 (12.5)	-	-	-	1 (3.1)	1 (2.9)	3 (3.7)	7 (6.0)
Artemether/lumefantrine	-	2 (7.7)	-	-	-	-	-	1 (0.9)
Other antimalarials	-	-	-	4 (8.2)	1 (3.1)	1 (2.9)	3 (3.7)	3 (2.6)
Vitamins	2 (25.0)	5 (19.2)	3 (33.3)	6 (12.2)	6 (18.8)	6(17.1)	20 (24.7)	22 (19.0)
Other non-antimalarials	4 (50.0)	7 (26.9)	2 (22.2)	11 (22.4)	2 (6.3)	6 (17.1)	13 (16.0)	23 (19.8)

### Treatment timeliness and cost

Survey respondents were also asked to state the time interval between the onset of the malaria symptoms and the time they sought treatment outside home. There were significant differences in treatment timeliness between areas. We found that 57.3% of urban caretakers and only 35.8% of rural caretakers sought treatment on the first day of illness onset (p<0.001). Among those, 19.8% of urban caretakers and only 9.5% of rural caretakers sought treatment immediately (p = 0.009).

**Fig 5:**
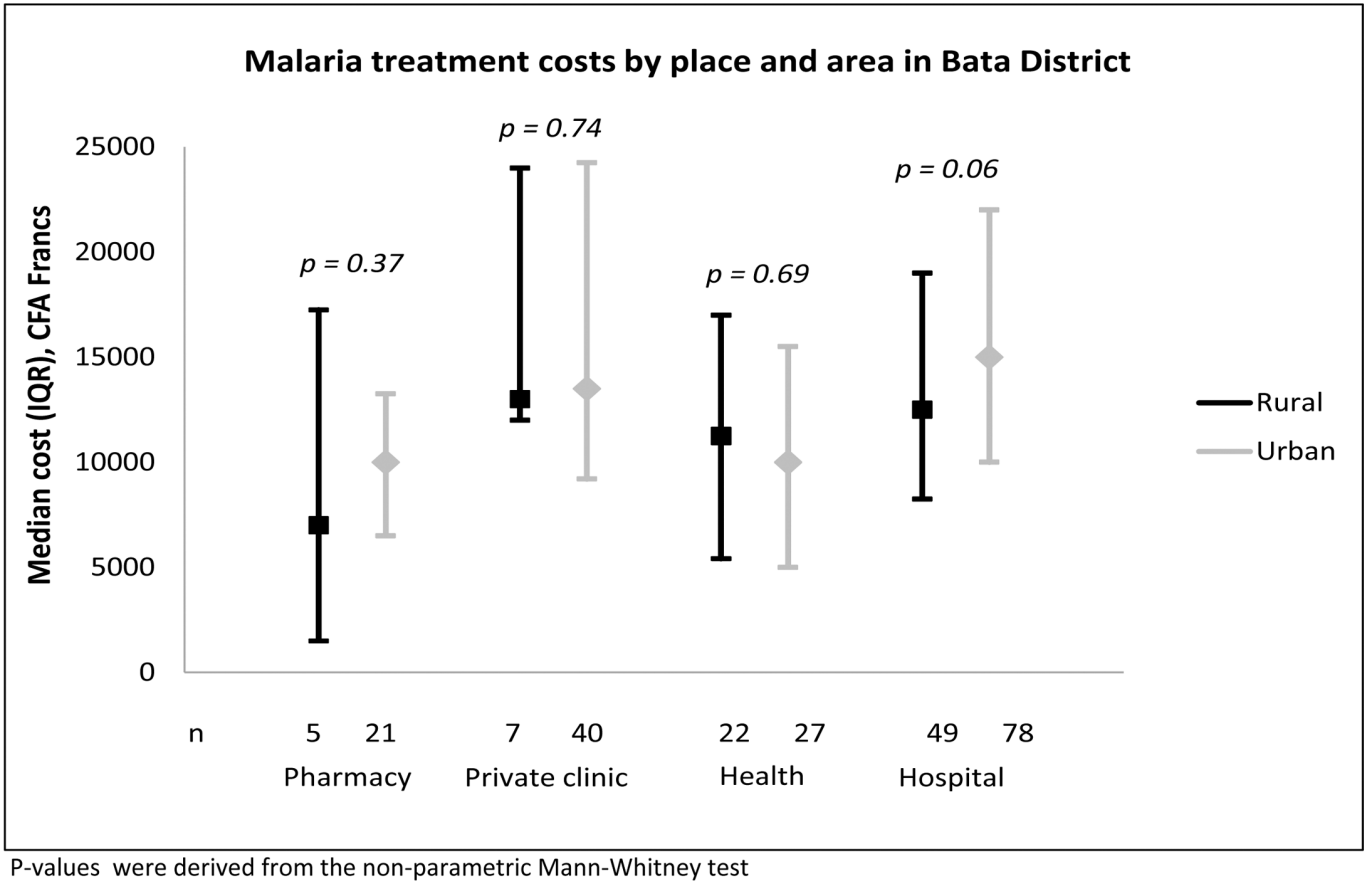
Malaria treatment costs by place and area in Bata District.

Of 428 children with reported malaria, 338 (79.0%) caretakers remembered that they paid something for the treatment. Fig 5 shows the median treatment costs by place and area. The median treatment costs were 12,000 (IQR: 5,375–16,000) CFA francs in rural areas and 12,500 (IQR: 8,000–20,000) CFA francs in urban Bata (p = 0.064). In both areas, treatments were least expensive at pharmacies and most expensive at hospitals. Treatment costs in both pharmacies and hospitals were lower to rural than to urban households, but the differences did not reach statistical significance. Only one urban caretaker remembered that the cost for the traditional healer was 58,000 CFA, which was more expensive than any other treatment.

## Discussion

This study offered several insights about the health-seeking behaviour of caregivers for children with reported malaria, the household diagnoses, the treatments administered and the treatment costs in the District of Bata, Equatorial Guinea. The marked differences between the rural and urban areas in population structures and access to treatment providers were in some extent present in the treatment seeking patterns of rural and urban caregivers.

In both rural and urban Bata households, fever was the main symptom associated with reported malaria in children, mostly reported alone, but it was also combined with other symptoms, like weakness, nausea, and headache. For many years, malaria has been considered the primary cause of all febrile illnesses in high-transmission settings [15]. This important relationship seemed to be established by Bata caretakers. Nevertheless, convulsions was the second most reported symptom alone in both rural and urban households. Elsewhere, convulsions were considered a key symptom associated with severe malaria, and its occurrence frequently led to a change in treatment actions [16,17]. However, this attitude was not apparent in Bata, because the occurrence of convulsions was not linked to a prompt or different pattern in seeking treatment. This behaviour might be reflecting a low perception of illness severity that should be further studied.

Up to three different lines of treatment were identified in Bata for a single malaria episode. Most household caregivers sought out more than one line of treatment for children with reported malaria starting at home and then seeking care elsewhere if the child’s condition did not improve [12,18,19]. The percentage of caregivers that sought a second line of treatment in the Bata District was higher than that observed in other African countries [20–22]. However, the proportion of caregivers that sought a third line of treatment was very low, particularly in urban Bata.

Home treatment was the first option for most caregivers in the Bata District. This option was chosen significantly more frequently in rural than in urban areas, possibly due to a perceived high treatment cost or due to geographic accessibility [12]. This behaviour was found in most African areas of high transmission [9,19,23,24], even in areas with widely available and accessible biomedical and health services [17].

Most drugs taken at home were antipyretics/analgesics, particularly in urban Bata. The use of traditional herbs for treating malaria at home is widespread in Africa [19], but in the Bata District, the use of herbs at home was less frequent than elsewhere [25]. Moreover, this practice was much more extensive in rural than in urban areas. This finding could be attributed to the low availability of conventional treatments in rural areas and the socioeconomic differences found between rural and urban caretakers. Rural caretakers were older and less literate than urban caretakers; thus, they may have more reliance in traditional treatments. However, the herbs, Nfoo and Eku, have been identified as Enanthiachiorantha and Aistoniaboonei [26], respectively. These plants are known to have antimalarial properties [27][28].

The high recovery rate reported of malaria cases that received only home treatment suggested that some cases might not have been malaria. This possibility points to the need to increase the availability of malaria diagnostic tools at the community level to avoid overtreatment [29,30]. Furthermore, caregivers that used traditional herbs at home reported that most children recovered; therefore, the efficacy and household dosage of *Nfoo* and *Eku* should be further explored.

Seeking care at health facility mainly comes after the failure of care at home [31] or caregivers might directly seek treatment from health facilities without initiating treatment at home [8]. In rural Bata, households mainly sought care in hospitals and health centres but in urban areas households mainly sought care in hospitals and private clinics. The relatively high use of hospitals in the Bata District may be associated, as in other countries, with the notion that such facility could better treat severe cases of malaria [22]. Community-based Health Workers (CHWs) were not mentioned by the households surveyed, even when they were available in rural Bata. Unfortunately, CHWs have not had access to diagnostic tools or treatments since 2011. Rural households in Bata were more likely to get treatment from their neighbours than urban households. This finding could be due to the homogeneous nature of rural residents [12]. The insignificant use of traditional healers for malaria treatment in Bata has been also reported elsewhere [5,24] related there with a low awareness of illness severity.

Artemether monotherapy was the most commonly prescribed antimalarial treatment throughout the Bata District. This therapy did not correspond to the first line treatment (AS+AQ) established by the national malaria treatment guidelines. The use of artemisinins in monotherapy involves serious risks because adherence to these relatively long treatment regimens is low [31]. This lack of adherence may result in late recrudescence or it may induce drug resistance. Moreover, the use of AS+AQ in the Bata District was very low. Health practitioners generally perceived AS+AQ as a drug with some side-effects; thus, some practitioners either refused to prescribe AS+AQ to patients or they reduced the drug dosage as a way of preventing or minimizing adverse effects [30]. A number of studies have documented case management practices in public health systems that did not adhere to national policies in sub-Saharan Africa [32]. Moreover, inappropriate prescription practices have also extended into the private sectors. These practices were found to be rooted in a sociocultural base to respond to the social expectations of the community which demanded injectable medications and wanted prompt treatment [17]. However, the EGMCI have to be aware that stock shortages occurred in Bata District may have made first line treatment compliance difficult.

The majority of Bata households waited for more than 24 hours before they sought outside treatment, but delays were longest in rural areas. Early recognition and diagnosis have been shown to be key factors in malaria control [32] reducing the chances of progression of the illness to severe disease [31].The difference in delay times might be explained by the proximity of health facilities in urban areas. In addition, rural households were poorest than urban ones and their inability to pay for health care may have caused delays in action, even though treatment costs were lower in rural than in urban areas [11].

The reported cost of malaria treatment in the Bata District varied from 200 to 58,000 CFA francs. The lower costs reported by rural Bata households may have reflected the fact that they received less healthcare than the urban households. Alternatively, it may reflect the use of lower level health care providers. Another possibility was that the rural households may not have been able to pay for all the drugs prescribed to them [12]. A formulation of economically viable and equitable drug policies would have the maximum impact on efforts to reduce the mortality and morbidity of malaria [33].

This study has some limitations. Firstly, the treatment-seeking behaviour registered was based on reported malaria thus some cases may not have been malaria. However, this would not have changed the reported behaviour of caregivers because they suspected it was truly malaria and acted accordingly. Secondly, there could be a problem of recall when the most recent malaria episode occurred long time back. However, we do not think this may influenced our results as we did not find significant differences in the main variables analysed according to the time length since the last malaria episode. Thirdly, although there could be considered difficult to properly identify some of the symptoms reported by caretakers in children less than one year old, as headache and nausea, these symptoms where reported only in children aged more than 8 months.

## Conclusions

Although rural and urban households in the Bata District recognized fever as the main symptom of malaria in children, differences existed in access to treatment and health-seeking responses to malaria. These findings point to important targets for maximizing the effects of the EGMCI programme. We found that delays in seeking treatment outside home, the type of malaria therapy received, and the treatment cost were the principal problems that need to be addressed. These issues are imbued with the notion that treatment failures are patient failures while we should be pressing to find ways to increase people’s capacity to access and complete effective treatment. Information, education and communication materials on possible actions to be taken by caretakers should be made fully available in both urban and rural areas [23].

Access to free diagnoses and treatments have been shown to be a major determinant in malaria control [34]. The available health care options, including proper home management, pharmacies, and health facilities, need to be reinforced in both urban and rural areas of the Bata District.

The low accomplishment of national treatment policies in the Bata District indicate the need for intensified interventions that target the community, health facilities, and drug vendors, to educate individuals on policy changes and the dangers of artemisinin monotherapy. Drug procurement and supply should be effectively managed to ensure access to the national first-line treatment as well as removal of ineffective drugs that should not be used to treat malaria. National treatment guidelines on drug utilization should be revised, and information about the correct application of drugs should be distributed to all the actors involved.

To reduce malaria morbidity and mortality in the area will require stressing the importance of prompt treatment within 24 hours of symptom onset and providing sufficient supplies of effective antimalarial drugs. In addition, improvements in malaria treatment skills in households and in health care workers are needed in both the public and private sectors.

## Supporting Information

S1 Table TS1. Symptoms mentioned by caretakers of children with reported malaria in Bata District. (DOCX)

S2 Table TS2. Drugs administered to children that received treatment at home in Bata District (DOC)
