# Correction: Rural-Urban Differences in Household Treatment-Seeking Behaviour for Suspected Malaria in Children at Bata District, Equatorial Guinea

**DOI:** 10.1371/journal.pone.0138518

**Published:** 2015-09-14

**Authors:** Maria Romay-Barja, Inma Jarrin, Policarpo Ncogo, Gloria Nseng, Maria Jose Sagrado, Maria A. Santana-Morales, Pilar Aparicio, Basilio Valladares, Matilde Riloha, Agustin Benito

The seventh author’s name is spelled incorrectly. The correct name is: Pilar Aparicio.
