# Supplementary material for: Rural-Urban Differences in Household Treatment-Seeking Behaviour for Suspected Malaria in Children at Bata District, Equatorial Guinea
Source: PLoS One. 2015 Aug 18;10(8):e0135887. doi: 10.1371/journal.pone.0135887 (PMC4540319; doi:10.1371/journal.pone.0135887)
Supplement: S1 Table — (DOCX) [file pone.0135887.s001.docx]

**TS1. Symptoms mentioned by caretakers of children with reported malaria in Bata District**

| Rural | | | | | | | |
| --- | --- | --- | --- | --- | --- | --- | --- |
|  | Children age | | | | | | |
|  |  | <1 year | % | 1 - 5 years | % | >5 years | % |
| Fever | No | 4 | 20.0 | 17 | 16.0 | 11 | 23.4 |
|  | Yes | 16 | 80.0 | 89 | 84.0 | 36 | 76.6 |
| Convulsions | No | 17 | 85.0 | 92 | 86.8 | 38 | 80.9 |
|  | Yes | 3 | 15.0 | 14 | 13.2 | 9 | 19.1 |
| Nausea | No | 19 | 95.0 | 85 | 80.2 | 43 | 91.5 |
|  | Yes | 1 | 5.0 | 21 | 19.8 | 4 | 8.5 |
| Headache | No | 20 | 100.0 | 99 | 93.4 | 37 | 78.7 |
|  | Yes | 0 | 0.0 | 7 | 6.6 | 10 | 21.3 |
| Weakness | No | 12 | 60.0 | 68 | 64.2 | 36 | 76.6 |
|  | Yes | 8 | 40.0 | 38 | 35.8 | 11 | 23.4 |
| Urban | | | | | | | |
|  |  | Children age | | | | | |
|  |  | <1 year | % | 1 - 5 years | % | >5 years | % |
| Fever | No | 3 | 7.3 | 20 | 13.8 | 2 | 2.9 |
|  | Yes | 38 | 92.7 | 125 | 86.2 | 67 | 97.1 |
| Convulsions | No | 40 | 97.6 | 130 | 89.7 | 67 | 97.1 |
|  | Yes | 1 | 2.4 | 15 | 10.3 | 2 | 2.9 |
| Nausea | No | 35 | 85.4 | 107 | 73.8 | 58 | 84.1 |
|  | Yes | 6 | 14.6 | 38 | 26.2 | 11 | 15.9 |
| Headache | No | 40 | 97.6 | 142 | 97.9 | 54 | 78.3 |
|  | Yes | 1 | 2.4 | 3 | 2.1 | 15 | 21.7 |
| Weakness | No | 27 | 65.9 | 95 | 65.5 | 52 | 75.4 |
|  | Yes | 14 | 34.1 | 50 | 34.5 | 17 | 24.6 |
