# Supplementary material for: Rural-Urban Differences in Household Treatment-Seeking Behaviour for Suspected Malaria in Children at Bata District, Equatorial Guinea
Source: PLoS One. 2015 Aug 18;10(8):e0135887. doi: 10.1371/journal.pone.0135887 (PMC4540319; doi:10.1371/journal.pone.0135887)
Supplement: S2 Table — (DOC) [file pone.0135887.s002.doc]

**TS2.Drugs administered to children that received treatment at home in Bata District**

| Rural | | | | | | | |
| --- | --- | --- | --- | --- | --- | --- | --- |
|  |  | Children age | | | | | |
|  |  | <1 year | % | 1 - 5 years | % | >5 | % |
| Paracetamol | No | 5 | 45.5 | 28 | 35.0 | 20 | 54.1 |
|  | Yes | 6 | 54.5 | 52 | 65.0 | 17 | 45.9 |
| Metamizole | No | 8 | 72.7 | 72 | 90.0 | 31 | 83.8 |
|  | Yes | 3 | 27.3 | 8 | 10.0 | 6 | 16.2 |
| Artemether | No | 11 | 100.0 | 75 | 93.8 | 34 | 91.9 |
|  | Yes | 0 | 0.0 | 5 | 6.3 | 3 | 8.1 |
| Artesunate | No | 11 | 100.0 | 79 | 98.8 | 35 | 94.6 |
|  | Yes | 0 | 0.0 | 1 | 1.3 | 2 | 5.4 |
| Sulfadoxine/pyremethamine | No | 11 | 100.0 | 80 | 100.0 | 36 | 97.3 |
|  | Yes | 0 | 0.0 | 0 | 0.0 | 1 | 2.7 |
| Chloroquine | No | 11 | 100.0 | 78 | 97.5 | 36 | 97.3 |
|  | Yes | 0 | 0.0 | 2 | 2.5 | 1 | 2.7 |
| Quinine | No | 11 | 100.0 | 77 | 96.3 | 35 | 94.6 |
|  | Yes | 0 | 0.0 | 3 | 3.8 | 2 | 5.4 |
| Traditional Herbs | No | 10 | 90.9 | 71 | 88.8 | 33 | 89.2 |
|  | Yes | 1 | 9.1 | 9 | 11.3 | 4 | 10.8 |
| Other antipyretics | No | 11 | 100.0 | 78 | 97.5 | 34 | 91.9 |
|  | Yes | 0 | 0.0 | 2 | 2.5 | 3 | 8.1 |
| Urban | | | | | | | |
|  |  | Children age | | | | | |
|  |  | <1 year | % | 1 - 5 years | % | >5 | % |
| Paracetamol | No | 7 | 35.0 | 30 | 28.8 | 11 | 21.6 |
|  | Yes | 13 | 65.0 | 74 | 71.2 | 40 | 78.4 |
| Metamizole | No | 19 | 95.0 | 85 | 81.7 | 44 | 86.3 |
|  | Yes | 1 | 5.0 | 19 | 18.3 | 7 | 13.7 |
| Artemether | No | 18 | 90.0 | 101 | 97.1 | 50 | 98.0 |
|  | Yes | 2 | 10.0 | 3 | 2.9 | 1 | 2.0 |
| Artesunate | No | 18 | 90.0 | 104 | 100.0 | 51 | 100.0 |
|  | Yes | 2 | 10.0 | 0 | 0.0 | 0 | 0.0 |
| Sulfadoxine/pyremethamine | No | 20 | 100.0 | 102 | 98.1 | 51 | 100.0 |
|  | Yes | 0 | 0.0 | 2 | 1.9 | 0 | 0.0 |
| Chloroquine | No | 20 | 100.0 | 103 | 99.0 | 51 | 100.0 |
|  | Yes | 0 | 0.0 | 1 | 1.0 | 0 | 0.0 |
| Quinine | No | 20 | 100.0 | 101 | 97.1 | 51 | 100.0 |
|  | Yes | 0 | 0.0 | 3 | 2.9 | 0 | 0.0 |
| Traditional Herbs | No | 19 | 95.0 | 101 | 97.1 | 51 | 100.0 |
|  | Yes | 1 | 5.0 | 3 | 2.9 | 0 | 0.0 |
| Other antipyretics | No | 18 | 90.0 | 101 | 97.1 | 49 | 96.1 |
|  | Yes | 2 | 10.0 | 3 | 2.9 | 2 | 3.9 |
